# Supplementary material for: Transcriptome Profiling of Maize (Zea mays L.) Leaves Reveals Key Cold-Responsive Genes, Transcription Factors, and Metabolic Pathways Regulating Cold Stress Tolerance at the Seedling Stage
Source: Genes (Basel). 2021 Oct 18;12(10):1638. doi: 10.3390/genes12101638 (PMC8535276; doi:10.3390/genes12101638)
Supplement: Supplementary file 1 [file genes-12-01638-s001.zip › genes-1360231-supplementary/SupplementaryFiguresTables/Figure S1.pdf]

genes

MDPI

Article

Transcriptome profiling of maize (*Zea mays* L.) leaves reveals key cold-responsive genes, transcription factors, and metabolic pathways regulating cold stress tolerance at the seedling stage

Joram Kiriga Waititu[1], Quan Cai[2], Ying Sun[1], Sun Yinglu[3], Congcong Li[1], Chunyi Zhang[1], Jun Liu[3], and Huan Wang[1,4] *

[1] Biotechnology Research Institute, Chinese Academy of Agricultural Sciences, Beijing 100081, China; joram.kiriga@gmail.com (J.K.W.); sunying0624@126.com (S.Y.); licongcong01@caas.cn, zhangchunyi@caas.cn (C.Z.); wanghuan@caas.cn (H.W.).

[2] Maize Research Institute, Heilongjiang Academy of Agricultural Sciences, Harbin 150086, China; cq6539@163.com (Q.C.).

[3] National Key Facility for Crop Resources and Genetic Improvement, Institute of Crop Science, Chinese Academy of Agricultural Sciences, Beijing 100081, China; sunny_5211995@sina.com (S.Y.L), liujun@caas.cn (J.L.).

[4] National Agricultural Science and Technology Center, Chengdu 610213, P.R. China; wanghuan@caas.cn (H.W.)

* Correspondence: wanghuan@caas.cn

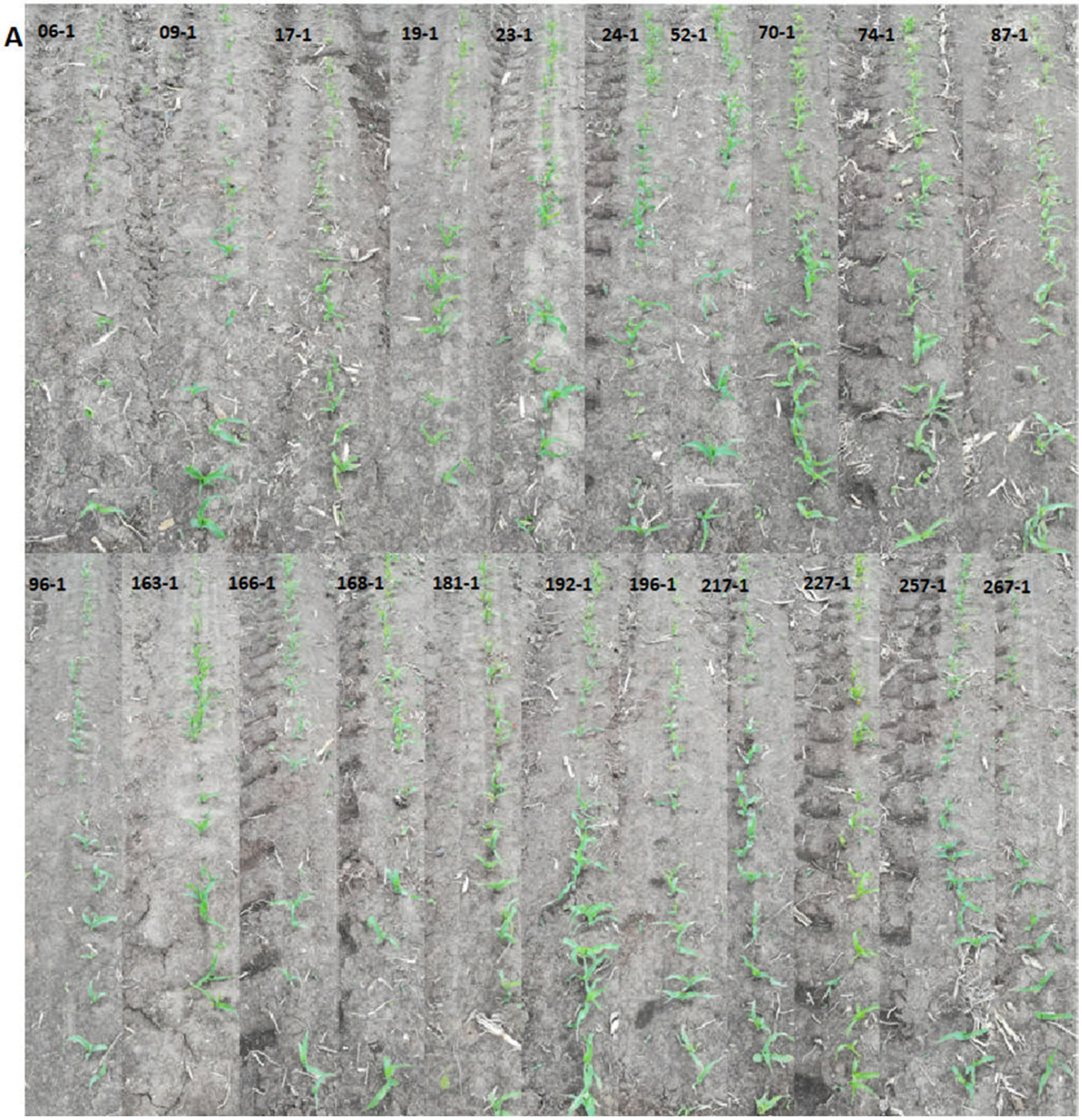
A
06-1
09-1
17-1
19-1
23-1
24-1
52-1
70-1
74-1
87-1
96-1
163-1
166-1
168-1
181-1
192-1
196-1
217-1
227-1
257-1
267-1

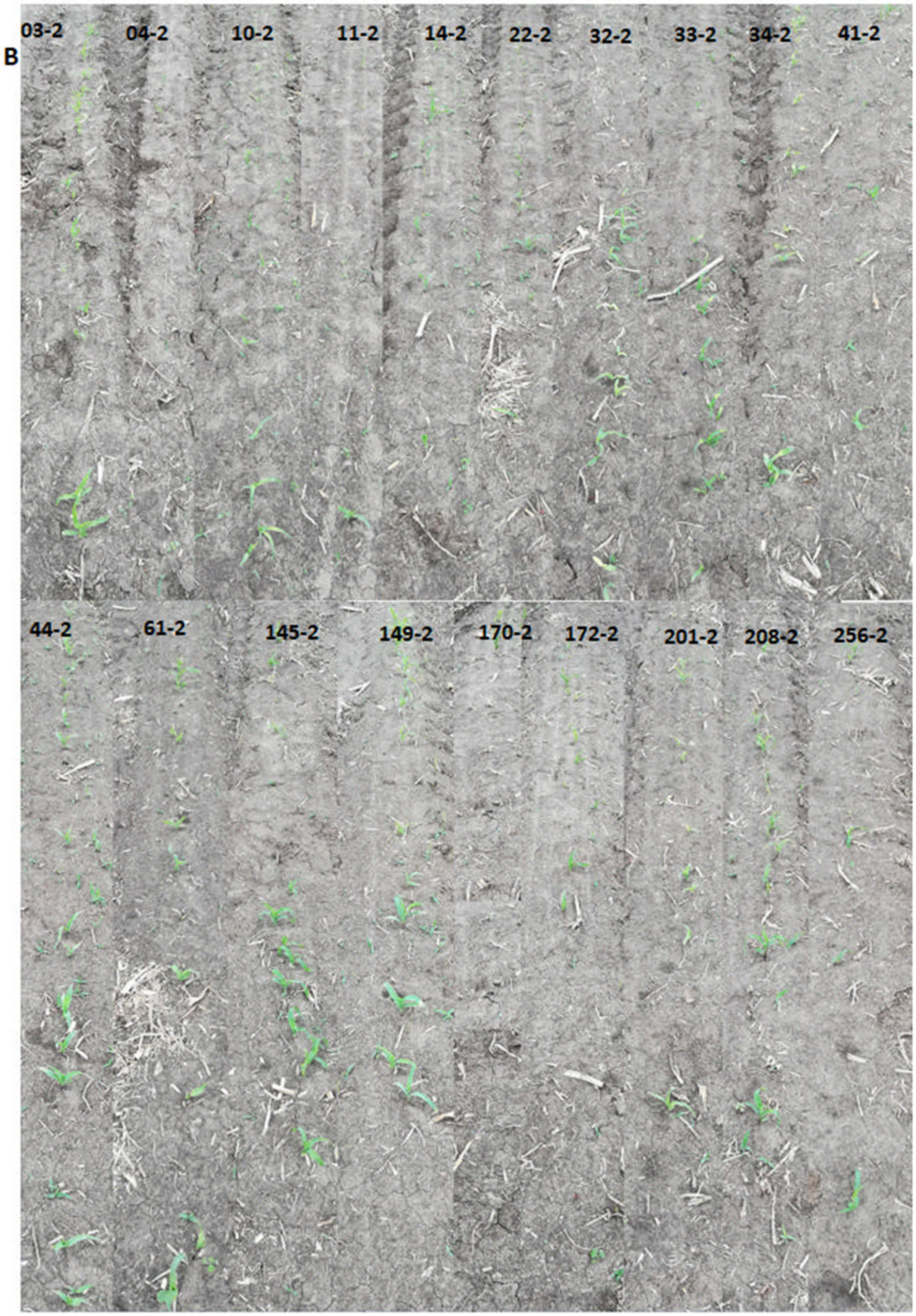

Figure S1. Phenotypic analysis of maize population under cold stress conditions (**A**) Tolerant lines (-1) maintained fully expanded green leaves, intact plant architecture, and high survival rates under cold stress (**B**) Sensitive lines (-2) displayed shriveled, curled, and yellowish coloured spots on the leaves under cold stress.
