# Supplementary material for: Transcriptome Profiling of Maize (Zea mays L.) Leaves Reveals Key Cold-Responsive Genes, Transcription Factors, and Metabolic Pathways Regulating Cold Stress Tolerance at the Seedling Stage
Source: Genes (Basel). 2021 Oct 18;12(10):1638. doi: 10.3390/genes12101638 (PMC8535276; doi:10.3390/genes12101638)
Supplement: Supplementary file 1 [file genes-12-01638-s001.zip › genes-1360231-supplementary/SupplementaryFiguresTables/Figure S2.pdf]

genes

MDPI

Article

Transcriptome profiling of maize (*Zea mays* L.) leaves reveals key cold-responsive genes, transcription factors, and metabolic pathways regulating cold stress tolerance at the seedling stage

Joram Kiriga Waititu[1], Quan Cai[2], Ying Sun[1], Sun Yinglu[3], Congcong Li[1], Chunyi Zhang[1], Jun Liu[3], and Huan Wang[1,4] *

[1] Biotechnology Research Institute, Chinese Academy of Agricultural Sciences, Beijing 100081, China; joram.kiriga@gmail.com (J.K.W.); sunying0624@126.com (S.Y.); licongcong01@caas.cn, zhangchunyi@caas.cn (C.Z.); wanghuan@caas.cn (H.W.).
[2] Maize Research Institute, Heilongjiang Academy of Agricultural Sciences, Harbin 150086, China; cq6539@163.com (Q.C.).
[3] National Key Facility for Crop Resources and Genetic Improvement, Institute of Crop Science, Chinese Academy of Agricultural Sciences, Beijing 100081, China; sunny_5211995@sina.com (S.Y.L), liujun@caas.cn (J.L.).
[4] National Agricultural Science and Technology Center, Chengdu 610213, P.R. China; wanghuan@caas.cn (H.W.)
* Correspondence: wanghuan@caas.cn

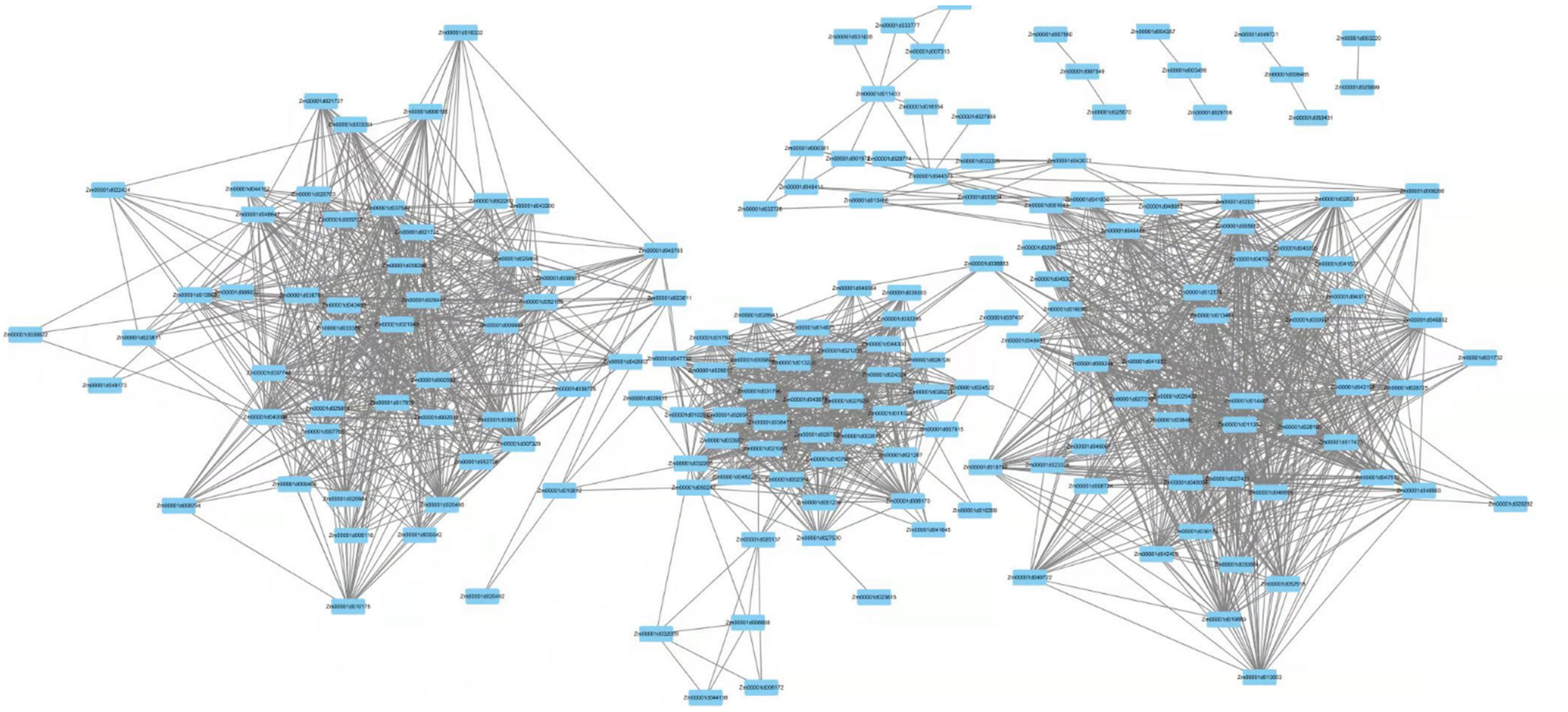

Figure S2. Co-expression network analysis of our DEGs from 24-tolerant and 22-sensitive maize inbred lines under cold stress conditions. The blue reactangle contains the Gene ID of the DEGs. For details about the crital genes, genes they regulate and expression pattern see supplementary material table S8.
