# Supplementary material for: Transcriptome Profiling of Maize (Zea mays L.) Leaves Reveals Key Cold-Responsive Genes, Transcription Factors, and Metabolic Pathways Regulating Cold Stress Tolerance at the Seedling Stage
Source: Genes (Basel). 2021 Oct 18;12(10):1638. doi: 10.3390/genes12101638 (PMC8535276; doi:10.3390/genes12101638)
Supplement: Supplementary file 1 [file genes-12-01638-s001.zip › genes-1360231-supplementary/SupplementaryFiguresTables/Table S3.pdf]

genes

MDPI

Article

Transcriptome profiling of maize (*Zea mays* L.) leaves reveals key cold-responsive genes, transcription factors, and metabolic pathways regulating cold stress tolerance at the seedling stage

Joram Kiriga Waititu[1], Quan Cai[2], Ying Sun[1], Sun Yinglu[3], Congcong Li[1], Chunyi Zhang[1], Jun Liu[3], and Huan Wang[1,4] *

[1] Biotechnology Research Institute, Chinese Academy of Agricultural Sciences, Beijing 100081, China; joram.kiriga@gmail.com (J.K.W.); sunying0624@126.com (S.Y.); licongcong01@caas.cn, zhangchunyi@caas.cn (C.Z.); wanghuan@caas.cn (H.W.).
[2] Maize Research Institute, Heilongjiang Academy of Agricultural Sciences, Harbin 150086, China; cq6539@163.com (Q.C.).
[3] National Key Facility for Crop Resources and Genetic Improvement, Institute of Crop Science, Chinese Academy of Agricultural Sciences, Beijing 100081, China; sunny_5211995@sina.com (S.Y.L), liujun@caas.cn (J.L.).
[4] National Agricultural Science and Technology Center, Chengdu 610213, P.R. China; wanghuan@caas.cn (H.W.)
* Correspondence: wanghuan@caas.cn

Table S3. Summary of RNA sequencing results for the forty-six maize seedling leaf samples.

Sample ID[1]	Total Reads (M)[2]	Total Bases (Gb)[3]	Q20[4]	Q30[5]	GC (%)[6]	Mapped reads (%)[7]
19NL06-1	45.86	6.88	96	92	53	86.09
19NL09-1	44.40	6.66	97	92	53	86.76
19NL17-1	46.46	6.97	95	90	52	81.13
19NL19-1	41.76	6.26	95	89	53	81.08
19NL23-1	44.84	6.73	96	92	53	85.37
19NL24-1	53.72	8.06	95	89	53	79.72
19NL52-1	40.06	6.01	93	86	52	78.45
19NL63-1	43.30	6.50	97	92	53	85.53
19NL70-1	46.42	6.96	96	92	52	84.51
19NL74-1	51.94	7.79	97	93	53	84.01
19NL87-1	41.79	6.27	94	88	53	80.14
19NL96-1	46.38	6.96	96	92	53	82.36
19NL153-1	56.86	8.53	97	92	53	83.55
19NL163-1	40.11	6.02	94	88	52	77.52
19NL166-1	53.13	7.97	97	92	53	83.20
19NL168-1	38.36	5.75	95	89	52	82.34
19NL181-1	54.56	8.18	96	92	53	82.74
19NL192-1	53.83	8.07	96	92	53	89.67
19NL196-1	45.18	6.78	97	92	52	89.81
19NL217-1	53.48	8.02	97	92	53	84.74
19NL222-1	47.91	7.19	95	90	52	79.76

19NL227-1	46.72	7.01	96	91	51	78.72
19NL257-1	45.95	6.89	96	92	53	87.90
19NL267-1	47.78	7.17	96	92	53	79.99
19NL22-2	52.60	7.89	96	92	52	81.75
19NL32-2	50.37	7.56	96	92	53	84.70
19NL33-2	51.80	7.77	96	92	53	83.39
19NL34-2	44.34	6.65	95	89	53	77.28
19NL41-2	60.24	9.04	96	92	53	84.27
19NL44-2	57.82	8.67	96	92	53	81.33
19NL03-2	50.70	7.61	95	89	52	79.41
19NL04-2	52.60	7.89	96	92	53	80.79
19NL10-2	54.47	8.17	97	92	53	83.38
19NL11-2	49.23	7.38	96	92	53	81.19
19NL14-2	47.49	7.12	94	88	52	79.70
19NL61-2	53.39	8.01	97	93	53	86.02
19NL145-2	43.01	6.45	97	93	52	83.36
19NL146-2	54.41	8.16	97	92	52	83.42
19NL149-2	46.63	6.99	95	89	52	80.75
19NL170-2	52.05	7.81	97	92	53	86.78
19NL172-2	57.94	8.69	95	89	52	79.37
19NL198-2	43.65	6.55	94	88	53	75.70
19NL199-2	40.28	6.04	95	89	52	79.69
19NL201-2	48.69	7.30	96	92	52	83.58
19NL208-2	56.79	8.52	97	92	53	83.82
19NL256-2	47.63	7.14	95	89	53	82.94

[1]Sample ID, maize inbred lines; cold-tolerant lines (-1), cold-susceptible lines (-2); [2]Total Reads (M), total clean reads in millions; [3]Total Bases (Gb), total bases in the data; [4]Q20, the percentage of the bases with a data mass value greater than or equal to 20; [5]Q30, the percentage of the bases with a data mass value greater than or equal to 30; [6]GC content, clean data GC content, that is, the percentage of G and C bases out of total bases in the data; [7]Mapped reads, the number of reads in the reference genome and the percentage in clean reads.
